# Polygenic risk for schizophrenia, disordered eating behaviours and body mass index in adolescents

**DOI:** 10.1192/bjp.2019.39

**Published:** 2019-07

**Authors:** Francesca Solmi, Marina Carbo Mascarell, Stanley Zammit, James B. Kirkbride, Glyn Lewis

**Affiliations:** 1 Sir Henry Wellcome Post-Doctoral Fellow, Division of Psychiatry, University College London, UK; 2 Division of Psychiatry, University College London, UK; 3 Professor of Psychiatric Epidemiology, Division of Psychological Medicine and Clinical Neurosciences, MRC Centre for Neuropsychiatric Genetics and Genomics, Cardiff University; and Professor of Psychiatry, Centre for Academic Mental Health, Bristol Medical School, University of Bristol, UK; 4 Reader in Epidemiology, Division of Psychiatry, University College London, UK; 5 Professor of Epidemiological Psychiatry, Division of Psychiatry, University College London, UK

**Keywords:** ALSPAC, schizophrenia, genetics, eating disorders

## Abstract

**Background:**

Recent studies suggest psychotic and eating disorders can be comorbid and could have shared genetic liability. However, this comorbidity has been overlooked in the epidemiological literature.

**Aims:**

To test whether polygenic risk scores (PRS) for schizophrenia are associated with disordered eating behaviours and body mass index (BMI) in the general population.

**Method:**

Using data from the Avon Longitudinal Study of Parents and Children and random-effects logistic and linear regression models, we investigated the association between PRS for schizophrenia and self-reported disordered eating behaviours (binge eating, purging, fasting and excessive exercise) and BMI at 14, 16 and 18 years.

**Results:**

Of the 6920 children with available genetic data, 4473 (64.6%) and 5069 (73.3%) had at least one disordered eating and one BMI outcome measurement, respectively. An s.d. increase in PRS was associated with greater odds of having binge eating behaviours (odds ratio, 1.36; 95% CI 1.16–1.60) and lower BMI (coefficient, −0.03; 95% CI, −0.06 to −0.01).

**Conclusions:**

Our findings suggest the presence of shared genetic risk between schizophrenia and binge eating behaviours. Intermediate phenotypes such as impaired social cognition and irritability, previously shown to be positively correlated in this sample with schizophrenia PRS, could represent risk factors for both phenotypes. Shared genetic liability between binge eating and schizophrenia could also explain higher rates of metabolic syndrome in individuals with schizophrenia, as binge eating could be a mediator of this association in drug-naïve individuals. The finding of an association between greater PRS and lower BMI, although consistent with existing epidemiological and genetic literature, requires further investigation.

**Declaration of interest:**

None.

The comorbidity of non-affective psychoses and eating disorders has received little attention in the literature. Nevertheless, both early accounts^1^ and more recent investigations^2^ suggest that disordered eating attitudes are common in individuals with schizophrenia, particularly binge eating and night-eating syndrome.^2^ Although some of these behaviours might be a consequence of pharmacological treatments,^3^ they have also been observed in drug-naïve individuals.^4^ A recent, large multi-country study has shown that participants with bulimia nervosa, binge eating disorder and anorexia nervosa had up to three times the odds of developing psychotic symptoms, and that those with psychotic symptoms have twice the odds of developing bulimia nervosa.^5^ Other studies have shown cross-sectional^6^ and longitudinal^7^ associations between psychotic experiences and disordered eating behaviours, including fasting, purging and binge eating, in both adults and adolescents. In line with these clinical and epidemiological findings, recent genome-wide association studies (GWAS) have identified a positive genetic correlation between schizophrenia and anorexia nervosa.^8^ Both conditions also share a negative genetic correlation with body mass index (BMI),^8^ reflecting low BMI as a clinical feature of anorexia nervosa and a risk factor for schizophrenia.^9^ Although large GWAS of bulimia nervosa and binge eating disorder do not yet exist, positive genetic correlations between schizophrenia and bipolar disorder and depression (both highly comorbid with bulimia nervosa and binge eating disorder) have been observed.^8^ Because disordered eating behaviours such as binge eating and purging can also occur in people with anorexia nervosa and studies have shown that eating disorder diagnoses are unstable,^10^ a shared genetic aetiology between schizophrenia and the entire eating disorder phenotypical spectrum is plausible. To date, however, no epidemiological study has directly investigated this hypothesis in the general population. Evidence of a shared genetic association between schizophrenia and eating disorders would support observational findings showing the co-occurrence of psychotic and eating disorders in the general population and highlight the need for clinical approaches that account for this possible comorbidity. It could also raise novel hypotheses for early-life intermediate phenotypes, which might be common across disorders and could provide potential targets for intervention. The aim of this study was therefore to investigate the presence of a common genetic liability across these disorders. To do this, we tested whether polygenic risk scores (PRS) for schizophrenia – a composite score capturing genetic susceptibility to the disorder – were associated with disordered eating behaviours frequently observed across eating disorder diagnoses (binge eating, purging, fasting and excessive exercise) and BMI, as low BMI is a distinct clinical feature of anorexia nervosa.

## Method

### Sample

We used data from the Avon Longitudinal Study of Parents and Children (ALSPAC), an ongoing birth cohort study of children born in Avon (Bristol, UK) between 1 April 1991 and 31 December 1992, who were alive at 1 year of age (*n* = 13 988, 99.5% of all live births). More details on the ALSPAC sample is available in previous publications^11^ and on the study website (www.bristol.ac.uk/alspac), which also contains details of all the available data in a fully searchable data dictionary (http://www.bris.ac.uk/alspac/researchers/data-access/data-dictionary/). This study includes children with available genetic data who had information on disordered eating behaviours on at least one time point across ages 14, 16 and 18 years. For twins, although it was not possible to distinguish whether they were monozygotic or dizygotic, we only retained the first born (twin A), as twins are non-independent observations. As sensitivity analyses, we further re-run our models including the second-born twin (twin ‘B’, *N* = 202) to explore if this resulted in any changes. We further restricted analyses to participants of White ethnicity as current PRS have sufficient discriminant validity in individuals of European ancestry.^12^ All participants gave their informed consent to participate in the study. The ALSPAC ethics and law committee and the local research ethics committees approved this study.

### Exposure

PRS are a continuous variable capturing the cumulative effect of risk alleles for a disorder in a given individual. Although PRS are not yet able to predict clinical presentations of disorders, they have increasingly been recognised as valid biomarkers and used to identify intermediate behavioural phenotypes or correlates of clinical disorders.^13^ The derivation of PRS for schizophrenia in this sample has been previously described in detail.^14^ Briefly, PRS were calculated using training set data from the second schizophrenia GWAS of the Psychiatric Genomics Consortium,^15^ the largest genetics consortium devoted to the study of psychiatric disorders.^13^

For each ALSPAC child, a total score was derived by adding the number of risk alleles in each single nucleotide polymorphism (SNP) (range, 0–2) weighted by the logarithm of its odds ratio for schizophrenia in the Psychiatric Genomics Consortium sample.^14^ In our main analyses, we used PRS derived from a set of SNPs selected using a *P*-value threshold of 0.05, as in previous ALSPAC studies,^14^ as this threshold best discriminates schizophrenia liability. To account for the need to balance true and false associations, in sensitivity analyses, we used scores derived from a broad range of *P*-value thresholds ranging from 1 × 10^−8^ to 0.5, as in previous investigations.^14^ PRS were standardised to achieve a continuous variable with a mean of 0 and an s.d. of 1. Squared PRS terms were also used to test for non-linear associations as well as quintiles of PRS.

### Outcomes

Data on disordered eating behaviours (binge eating, fasting, purging and excessive exercise) in the previous 12 months was self-reported in postal questionnaires by adolescents at approximately 14, 16 and 18 years of age, using a modified version of the Youth Risk Behaviour Survey.^16^ We considered each of the disordered eating behaviours under investigation as present (yes/no) if they had occurred at least once in the previous year. Binge eating was defined as having ever eaten large amounts of food in a short period of time while usually experiencing a sense of loss of control over the amount of food eating. We defined purging adolescents as those who had ever self-induced vomit or used laxatives for weight loss, and fasting adolescents as those who had ever fasted for an entire day to lose weight. We considered excessive exercise as exercising frequently for weight loss even when sick, or finding it difficult to do school work (age 14 years) or feeling guilty when not exercising (age 16 and 18 years), as these dimensions (as opposed to amount of exercise undertaken, for instance) have been shown to better capture eating disorder psychopathology.^17^ Objective measurements of height and weight were collected in clinic visits when adolescents were approximately 13, 15 and 17 years of age. From these measures we derived a continuous standardised measure of BMI, which was standardised at ages 13 and 15 years by age- and gender-specific *z*-scores derived from the updated Stata package *zanthro*,^18^ as recommended in the literature.^19^

### Confounders

In our analyses, we accounted for population stratification by conducting principal component analyses and adjusting our models for population principal components as covariates. We also described our sample in relation to a number of child and family characteristics, including child's gender, maternal age, marital status (single/married/divorced, widowed or separated) and education (compulsory/non-compulsory education).

### Data analysis

To take in to account the repeated nature of our outcome measurements, we used random-effects logistic and linear multilevel regressions (Stata commands *xtlogit* and *xtreg*, respectively), which allowed us to model repeated follow-up time points nested within individuals. In our models, we further added a linear variable for age (age 14, 16 and 18 years) to model the effect of time, as well as population principal components to account for population stratification. We tested for non-linear associations with a quadratic term for PRS. To further explore non-linearity and dose-response patterns of these associations, we divided PRS scores in quintiles and re-run our models with this variable as our main exposure. As sensitivity analyses, we tested for these associations using continuous measures of PRS derived from risk alleles associated with schizophrenia at different *P*-value thresholds, as previously done.^14^ We conducted all analyses on Stata version 13 for Windows.^20^

## Results

### Sample

In total, 6920 children had available genetic data. Of these, 4473 (64.6%) and 5069 (73.3%) had data available at least at one time point on all disordered eating variables and one BMI outcome measurement, respectively. Across outcome measurements, between 26.8% (*n* = 1851, BMI) and 34.5% (*n* = 2387, binge eating) did not have any outcome data available, and between 24.8% (*n* = 1718, excessive exercise) and 42.8% (*n* = 2961, BMI) had data at all three time points. Proportions of children with two time points of data available varied between 16.6% (*n* = 1151, BMI) and 21.0% (*n* = 1454, excessive exercise), and that of children with only one time point available ranged between 13.8% (*n* = 957, BMI) and 19.4% (*n* = 1345, fasting and excessive exercise; data available from authors).

As shown in Table 1, just over half of our sample were girls (54.9%) and most children had a married mother (84.3%) who had completed compulsory education (52.2%) and was, on average, 29.4 years of age.

**Table 1:** Sample characteristics and polygenic risk score distribution

	*N* (%)	Polygenic risk score, mean (s.d.)
Gender		
Male	2019 (45.14%)	−0.04 (1.00)
Female	2454 (54.86%)	−0.05 (1.00)
Maternal education		
Compulsory	2327 (52.15%)	−0.08 (0.98)
Non-compulsory	2135 (47.85%)	−0.01 (1.01)
Marital status		
Single	496 (11.20%)	0.02 (1.01)
Married	3733 (84.27%)	−0.07 (1.00)
Divorced, separated, widowed	201 (4.53%)	0.09 (0.98)
	Mean (s.d.)	Correlation
Maternal age	29.44 (4.45)	0.05

Table 2 shows the prevalence of disordered eating behaviours at age 14, 16 and 18 years. In line with the known epidemiology of eating disorders, prevalence of all behaviours was relatively low, but increased over time. All disordered eating behaviours were also more prevalent in girls compared with boys at all time points (data shown in Supplementary Table 1 available at https://doi.org/10.1192/bjp.2019.39).

**Table 2:** Twelve-month prevalence of disordered eating behaviours at ages 14, 16 and 18 years

Time points	Disordered eating behaviours			
	Binge eating,^a^ *n* (%)	Purging,^a^ *n* (%)	Fasting,^a^ *n* (%)	Excessive exercise,^a^ *n* (%)
Age 14 years	*N* = 3899 48 (1.2%)	*N* = 3879 62 (1.6%)	*N* = 3875 240 (6.2%)	*N* = 3860 80 (2.1%)
Age 16 years	*N* = 3296 124 (3.8%)	*N* = 3282 197 (6.0%)	*N* = 3284 439 (13.4%)	*N* = 3338 88 (2.7%)
Age 18 years	*N* = 2232 97 (4.4%)	*N* = 2207 141 (6.4%)	*N* = 2228 261 (11.5%)	*N* = 2220 83 (3.7%)

a Sample sizes vary slightly across outcomes at each time point because of different patterns of missing data across questions used to derive them.

### Schizophrenia PRS and disordered eating behaviours

As shown in Table 3, in logistic regression models, we found strong evidence that a 1 s.d. increase in PRS for schizophrenia was associated with a 1.36 increase in the odds of binge eating (95% CI 1.16–1.60, *P* < 0.0001). Greater PRS were not associated with purging behaviours (odds ratio, 1.03; 95% CI 0.88–1.21; *P* = 0.71), fasting (odds ratio, 1.02; 95% CI 0.91–1.15; *P* = 0.73) or excessive exercise (odds ratio, 1.09; 95% CI 0.95–1.26; *P* = 0.19). Finally, an s.d. increase in PRS was associated with a −0.03 s.d. decrease in BMI (95% CI −0.06 to −0.01; *P* = 0.02), although evidence of this association was moderate to weak. We did not find any evidence of non-linear relationships when using quadratic PRS terms.

**Table 3:** Random-effects logistic and linear regression model results

Outcomes	PRS odds ratio (95% CI)	*P*-value
Binge eating^a^	1.36 (1.16–1.60)	<0.0001
Purging^a^	1.03 (0.88–1.21)	0.71
Fasting^a^	1.02 (0.91–1.15)	0.73
Excessive exercise^a^	1.09 (0.95–1.26)	0.19
Body mass index^b^	−0.03 (−0.06 to −0.01)	0.02

PRS, polygenic risk score.

a *N* = 4473.

b *N* = 5069.

When we investigated these associations using quintiles of PRS, we observed that children in the top three quintiles had the highest odds of binge eating and lower BMI (see Table 4).

**Table 4:** Multilevel logistic and linear regression models for the association between quintiles of polygenic risk scores for schizophrenia and disordered eating and body mass index

	Binge eating,^a^ odds ratio (95% CI)	Purging,^a^ odds ratio (95% CI)	Fasting,^a^ odds ratio (95% CI)	Excessive exercise,^a^ odds ratio (95% CI)	Body mass index,^b^ coefficient (95% CI)
First quintile	Reference	Reference	Reference	Reference	Reference
Second quintile	0.90 (0.51–1.58) *P* = 0.70	1.14 (0.69–1.88) *P* = 0.61	1.00 (0.70–1.44) *P* = 0.99	0.96 (0.61–1.50) *P* = 0.86	−0.08 (−0.16 to 0.01) *P* = 0.10
Third quintile	1.68 (1.00–2.83) *P* = 0.05	0.94 (0.56–1.58) *P* = 0.81	0.95 (0.66–1.37) *P* = 0.77	0.97 (0.62–1.52) *P* = 0.91	−0.10 (−0.19 to −0.01) *P* = 0.03
Fourth quintile	1.77 (1.05–2.98) *P* = 0.03	1.33 (0.80–2.19) *P* = 0.27	1.34 (0.94–1.92) *P* = 0.11	1.14 (0.73–1.76) *P* = 0.57	−0.08 (−0.17 to 0.01) *P* = 0.06
Fifth quintile	2.37 (1.41–3.96) *P* = 0.001	1.16 (0.69–1.96) *P* = 0.57	1.05 (0.72–1.52) *P* = 0.80	1.19 (0.67–1.87) *P* = 0.44	−0.12 (−0.21 to −0.03) *P* = 0.01

a *N* = 4473.

b *N* = 5069.

#### Sensitivity analyses

When we used PRS derived from SNPs with different *P*-value thresholds in the GWAS sample, our findings were overall consistent, although associations became more prominent at larger *P*-value thresholds (findings shown in Supplementary Figs 1 and 2). Including the other twin (twin B) in our sample did not alter our findings in relation to binge eating (odds ratio, 1.38; 95% CI 1.17–1.62; *P* < 0.0001) or BMI (coefficient, −0.03; 95% CI −0.06 to −0.01; *P* = 0.026; data available from authors).

## Discussion

Using a large prospective cohort of British children, this study showed an association between genetic risk of schizophrenia and disordered eating behaviours. In particular, we found that higher schizophrenia PRS were associated with greater odds of binge eating, a behaviour that is central to the diagnoses of bulimia nervosa and binge eating disorder, but that can also occur in individuals with anorexia nervosa of the binge-purge type. We also observed some evidence of an association between greater schizophrenia PRS and lower BMI. These patterns of associations were largely consistent across a range of PRS training-set *P*-value thresholds.

### Strengths and limitations

This study has a number of strengths. We used a large general population sample of adolescents whose disordered behaviours were self-reported across adolescence, thus avoiding sampling biases often associated with clinically diagnosed cases. The PRS we used were derived using findings from the largest schizophrenia GWAS to date.^15^ Our BMI measure was obtained from objective measurements, thus avoiding reporting biases, and standardised using age and gender growth curves. The detailed characterisation of the ALSPAC sample allowed us to investigate which specific disordered eating phenotypes might be underpinned by shared genetic liability with psychotic disorders. The use of repeated measurements across adolescence also allowed us to fully exploit the richness of the data-set, minimise the role of missing data and account for outcome clustering within individuals.

Several limitations should nevertheless be noted. Our disordered eating definitions were broad and some of the participants might have experienced these behaviours at a very low threshold. However, previous evidence in this sample has shown that even adolescents with broad disordered eating presentations experienced comorbidity and long-term outcomes^21^ comparable with those observed in full diagnoses. This finding is consistent with the idea that eating disorders, like other psychiatric disorders, occur on a continuum of severity and suggest that these definitions can be useful for future studies investigating the aetiology of and outcomes associated with these conditions. Our sample was also characterised by a notable degree of attrition, which was predicted by greater schizophrenia PRS. It is possible that collider (i.e. selection) bias, which occurs if selection into the study is conditional on the exposure and outcome measured, could have induced or prevented the observation of some of the associations under study. The PRS measure that we used was derived in an adult population and might not capture genetic liability to early-onset cases of schizophrenia. However, this measure has been extensively used to explore intermediate phenotypes in general population samples, hence our approach aligns with that of previous investigations.^13^^,^^14^ Finally, although they were derived from a large discovery data-set, it has been shown that the PRS measure we used only explain around 7% of the variance in schizophrenia.^15^ Hence, we cannot exclude that some of the associations that we did not observe were due to the limited predictive value of our exposure measure.

### Comparisons with previous literature and interpretation of the findings

Although this is the first study to show an association between schizophrenia PRS and binge eating, this finding is consistent with those of previous epidemiological investigations in clinical and general population samples.^1^^,^^5^^,^^6^ For instance, McGrath *et al* have recently shown that individuals with lifetime psychotic symptoms had two to four times the odds of also experiencing bulimia nervosa and binge eating disorder, respectively, two eating disorders for whose diagnosis binge eating is a central criterion.^5^ A cross-sectional study of UK adults found that those who experience loss of control over eating (a symptom of binge eating) were more likely to report auditory hallucinations and other psychotic experiences.^6^ In this sample, psychotic experiences at age 13 years were associated with greater disordered eating behaviours, including fasting and binge eating, at age 18 years.^7^ Clinical studies also found that individuals with non-affective psychoses had greater disordered eating, including binge eating,^4^ and that individuals with eating disorders, and in particular those who binge eat,^22^ display greater comorbidity with psychotic illnesses^1^ or psychotic symptoms.^22^

Shared genetic liability between schizophrenia, bipolar disorder and major depressive disorder^8^ could explain our findings. Epidemiological studies in clinical^23^ and general population^24^ samples have highlighted a high comorbidity between bipolar disorder and bulimia nervosa and binge eating disorder. However, the cross-sectional or retrospective designs of these studies have so far limited aetiological inferences about the temporality of these associations or presence of shared risk factors. By investigating the association between schizophrenia PRS and binge eating in our longitudinal birth cohort, our findings suggest that the observed comorbidity between these conditions could be partially explained by shared genetic liability, although potential risk pathways remain unclear.

Previous studies have also shown that higher schizophrenia PRS are associated with poorer social understanding and greater irritability,^25^ domains that appear to be impaired in individuals with eating disorders^26^^,^^27^ and could therefore represent intermediate phenotypes shared by these conditions. Although the majority of the literature has focused on anorexia nervosa,^28^ a recent systematic review has shown that those with broader disordered eating behaviours^26^ also show deficits in social cognition, and greater autistic traits. To date, these studies have been solely conducted cross-sectionally,^26^ making it difficult to infer whether these cognitive deficits traits emerge before or after the onset of eating disorders. However, a recent study has shown that children of mothers with binge-purge behaviours had poorer social communication and emotion recognition skills, which the authors suggested could represent intermediate phenotypes in the intergenerational transmission of eating disorders.^29^

Irritability in early life, shown to be associated in this sample with greater genetic risk of schizophrenia,^25^ could also represent an early risk factor for binge eating. Literature on irritability in childhood as a risk factor for eating disorders is limited, although clinical accounts report that individuals with eating disorders show greater irritability.^27^ Difficulties in regulating negative emotions have also been cross-sectionally associated with loss-of-control eating in adolescence,^30^ mirroring findings of adult populations showing that dysregulated eating behaviours can serve to regulate immediate emotional states.^31^ Longitudinal studies investigating the association between social cognition and irritability in childhood and disordered eating in the general population are therefore warranted, as these could be transdiagnostic markers of risk across multiple phenotypes.

The observed association between PRS for schizophrenia and binge eating also raises a number of hypotheses regarding the observed presence of similar metabolic abnormalities shared by people with psychotic illnesses and binge eating disorder. In people with psychotic disorders, metabolic abnormalities may arise for several reasons, including use of antipsychotics. Nevertheless, impaired glycaemic control and insulin resistance have also been shown in adolescents with non-clinical psychotic experiences,^32^ as well as in drug-naïve individuals with schizophrenia and their first-degree relatives.^33^ Similar metabolic abnormalities are observed among individuals with binge eating disorder.^34^ Although more research is needed to disentangle temporal associations, our findings lend themselves to two possible interpretations, which will require further investigation. First, it is possible that metabolic abnormalities could represent shared risk factors for both of these phenotypes. Second, binge eating could represent a mediator in the association between psychotic experiences and dysglycaemia. This hypothesis is supported by recent findings showing that insulin resistance measured at age 9 years was not associated with greater psychotic experiences at age 18 years, but those with psychotic experiences at 18 years had, cross-sectionally, greater insulin resistance.^32^ In turn, another study found that psychotic experiences at age 13 years predicted greater binge eating behaviours at age 18 years,^7^ suggesting that the latter could be a contributing factor in the onset of metabolic abnormalities.

In light of the observed association between PRS and binge eating, often associated with greater BMI, our finding that schizophrenia PRS predict lower BMI is difficult to interpret. This association, however, has been previously observed,^35^ and is in line with findings of genetic studies showing an inverse correlation between schizophrenia and BMI,^8^ and with those of epidemiological investigations showing that low BMI is a risk factor for schizophrenia.^9^ The pathophysiological mechanisms underpinning the association between genetic liability for schizophrenia and low BMI are unclear, particularly as clinically, overweight and obesity are more frequently observed in individuals with psychotic illnesses.^36^ Environmental factors associated with increased genetic risk (i.e. deprivation) could explain low BMI before disease onset, and greater BMI could be a result of medication use after disease onset. It is also possible that binge eating behaviours could contribute to greater BMI through the life course, so that emergence of binge eating in adolescence could partially contribute to minimising any existing differences in BMI. Another hypothesis is that genetic risk for schizophrenia leads to both binge eating and low BMI via different risk pathways.

To our knowledge, this is the first study to show an association between polygenic risk for schizophrenia and disordered eating behaviours. Although replication of these results is needed, we find that PRS are associated with greater risk of binge eating behaviours. Future research should investigate the presence of shared intermediate phenotypes across these disorders, including the role of metabolic abnormalities, to improve early identification of at-risk children. From a clinical viewpoint, these findings further highlight the need to consider the co-occurrence of disordered eating behaviours, particularly binge eating, in individuals with psychotic illnesses, as this might account for the weight gain observed in this population.

## Supplementary material

For supplementary material accompanying this paper visit https://doi.org/10.1017/10.1192/bjp.2019.39. click here to view supplementary material

## References

[1] Striegel-Moore RH, Garvin V, Dohm FA, Rosenheck RA. Eating disorders in a national sample of hospitalized female and male veterans: detection rates and psychiatric comorbidity. Int J Eat Disord 1999; 25: 405–14. 10202651 10.1002/(sici)1098-108x(199905)25:4<405::aid-eat5>3.0.co;2-f

[2] Kouidrat Y, Amad A, Lalau J-D, Loas G. Eating disorders in schizophrenia: implications for research and management. Schizophr Res Treatment 2014; 2014: 791573. 25485152 10.1155/2014/791573 PMC4251071

[3] Theisen FM, Linden A, König IR, Martin M, Remschmidt H, Hebebrand J. Spectrum of binge eating symptomatology in patients treated with clozapine and olanzapine. J Neural Transm 2003; 110: 111–21. 12541016 10.1007/s00702-002-0792-6

[4] Fawzi MH, Fawzi MM. Disordered eating attitudes in Egyptian antipsychotic naive patients with schizophrenia. Compr Psychiatry 2012; 53: 259–68. 21640339 10.1016/j.comppsych.2011.04.064

[5] McGrath JJ, Saha S, Al-Hamzawi A, Andrade L, Benjet C, Bromet EJ, The bidirectional associations between psychotic experiences and DSM-IV mental disorders. Am J Psychiatry 2016; 173: 997–1006. 26988628 10.1176/appi.ajp.2016.15101293 PMC5175400

[6] Koyanagi A, Stickley A, Haro JM. Psychotic-like experiences and disordered eating in the English general population. Psychiatry Res 2016; 241: 26–34. 27152907 10.1016/j.psychres.2016.04.045

[7] Solmi F, Melamed D, Lewis G, Kirkbride JB. Longitudinal associations between psychotic experiences and disordered eating behaviours in a general population sample of adolescents. Lancet Child Adolesc Heal 2018; 2: 591–9. 10.1016/S2352-4642(18)30180-9 PMC6054050 30119718

[8] Bulik-Sullivan B, Finucane HK, Anttila V, Gusev A, Day FR, Loh P-R, An atlas of genetic correlations across human diseases and traits. Nat Genet 2015; 47: 1236–41. 26414676 10.1038/ng.3406 PMC4797329

[9] Sørensen HJ, Gamborg M, Sørensen TIA, Baker JL, Mortensen EL. Childhood body mass index and risk of schizophrenia in relation to childhood age, sex and age of first contact with schizophrenia. Eur Psychiatry 2016; 34: 64–9. 26967349 10.1016/j.eurpsy.2016.01.2425

[10] Schaumberg K, Jangmo A, Thornton LM, Birgegård A, Almqvist C, Norring C, Patterns of diagnostic transition in eating disorders: a longitudinal population study in Sweden. Psychol Med 2018: 1–9. 10.1017/S0033291718001472 PMC6788452 29911514

[11] Boyd A, Golding J, Macleod J, Lawlor D, Fraser A, Henderson J, Cohort profile: the ‘children of the 90s’–the index offspring of the Avon Longitudinal Study of Parents and Children. Int J Epidemiol 2013; 42: 111–27. 22507743 10.1093/ije/dys064 PMC3600618

[12] Vassos E, Di Forti M, Coleman J, Iyegbe C, Prata D, Euesden J, An examination of polygenic score risk prediction in individuals with first-episode psychosis. Biol Psychiatry 2017; 81: 470–7. 27765268 10.1016/j.biopsych.2016.06.028

[13] Sullivan PF, Agrawal A, Bulik CM, Andreassen OA, Børglum AD, Breen G, Psychiatric genomics: an update and an Agenda. Am J Psychiatry 2018; 175: 15–27. 28969442 10.1176/appi.ajp.2017.17030283 PMC5756100

[14] Jones HJ, Stergiakouli E, Tansey KE, Hubbard L, Heron J, Cannon M, Phenotypic manifestation of genetic risk for schizophrenia during adolescence in the general population. JAMA Psychiatry 2016; 73: 221. 26818099 10.1001/jamapsychiatry.2015.3058 PMC5024747

[15] Schizophrenia Working Group of the Psychiatric Genomics Consortium. Biological insights from 108 schizophrenia-associated genetic loci. Nature 2014; 511: 421–7. 25056061 10.1038/nature13595 PMC4112379

[16] Kann L, Warren CW, Harris WA, Collins JL, Williams BI, Ross JG, Youth risk behavior surveillance–United States, 1995. MMWR CDC Surveill Summ 1996; 45: 1–84. 8841032

[17] Mond JM, Hay PJ, Rodgers B, Owen C. An update on the definition of ‘excessive exercise’ in eating disorders research. Int J Eat Disord 2006; 39: 147–53. 16231344 10.1002/eat.20214

[18] Vidmar SI, Cole TJ, Pan H. Standardizing anthropometric measures in children and adolescents with functions for egen: update. Stata J 2013; 13: 366–78.

[19] Cole TJ, Bellizzi MC, Flegal KM, Dietz WH. Establishing a standard definition for child overweight and obesity worldwide: international survey. BMJ 2000; 320: 1240–3. 10797032 10.1136/bmj.320.7244.1240 PMC27365

[20] StataCorp. *Stata Statistical Software: Release 13* StataCorp, 2013 (https://www.stata.com/).

[21] Micali N, Solmi F, Horton NJ, Crosby RD, Eddy KT, Calzo JP, Adolescent eating disorders predict psychiatric, high-risk behaviors and weight outcomes in young adulthood. J Am Acad Child Adolesc Psychiatry 2015; 54: 652–9.e1. 26210334 10.1016/j.jaac.2015.05.009 PMC4515576

[22] Aragona M. Psychotic phenomena in binge eating disorder: an exploratory MMPI-2 study. Arch Psychiatry Psychother 2015; 17: 13–20.

[23] McElroy SL, Frye MA, Hellemann G, Altshuler L, Leverich GS, Suppes T, Prevalence and correlates of eating disorders in 875 patients with bipolar disorder. J Affect Disord 2011; 128: 191–8. 20674033 10.1016/j.jad.2010.06.037

[24] Hudson JI, Hiripi E, Pope HG Jr, Kessler RC. The prevalence and correlates of eating disorders in the national comorbidity survey replication. Biol Psychiatry 2007; 61: 348–58. 16815322 10.1016/j.biopsych.2006.03.040 PMC1892232

[25] Riglin L, Collishaw S, Richards A, Thapar AK, Maughan B, O'Donovan MC, Schizophrenia risk alleles and neurodevelopmental outcomes in childhood: a population-based cohort study. Lancet Psychiatry 2017; 4(1): 57–62. 27932233 10.1016/S2215-0366(16)30406-0

[26] Christensen SS, Bentz M, Clemmensen L, Strandberg-Larsen K, Olsen EM. Disordered eating behaviours and autistic traits-Are there any associations in nonclinical populations? A systematic review. Eur Eat Disord Rev 2019; 27: 8–23. 10.1002/erv.2627 30058191

[27] Raffi AR, Rondini M, Grandi S, Fava GA. Life events and prodromal symptoms in bulimia nervosa. Psychol Med 2000; 30: 727–31. 10.1017/s0033291799002019 10883727

[28] Westwood H, Eisler I, Mandy W, Leppanen J, Treasure J, Tchanturia K. Using the autism-spectrum quotient to measure autistic traits in anorexia nervosa: a systematic review and meta-analysis. J Autism Dev Disord 2016; 46: 964–77. 26542816 10.1007/s10803-015-2641-0 PMC4746216

[29] Kothari R, Barona M, Treasure J, Micali N. Social cognition in children at familial high-risk of developing an eating disorder. Front Behav Neurosci 2015; 9: 1–17. 26300753 10.3389/fnbeh.2015.00208 PMC4528178

[30] Goldschmidt AB, Lavender JM, Hipwell AE, Stepp SD, Keenan K. Emotion regulation and loss of control eating in community-based adolescents. J Abnorm Child Psychol 2017; 45: 183–91. 27040409 10.1007/s10802-016-0152-x PMC5050053

[31] Macht M, Simons G. Emotions and eating in everyday life. Appetite 2000; 35: 65–71. 10896762 10.1006/appe.2000.0325

[32] Perry BI, Upthegrove R, Thompson A, Marwaha S, Zammit S, Singh SP, Dysglycaemia, inflammation and psychosis: findings from the UK ALSPAC birth cohort. Schizophr Bull 2018, in press. 10.1093/schbul/sby040 PMC6403055 29635418

[33] Chadda R, Ramshankar P, Deb K, Sood M. Metabolic syndrome in schizophrenia: differences between antipsychotic-naïve and treated patients. J Pharmacol Pharmacother 2013; 4: 176. 23960422 10.4103/0976-500X.114596 PMC3746300

[34] Succurro E, Segura-Garcia C, Ruffo M, Caroleo M, Rania M, Aloi M, Obese patients with a binge eating disorder have an unfavorable metabolic and inflammatory profile. Medicine (Baltimore) 2015; 94: e2098. 26717356 10.1097/MD.0000000000002098 PMC5291597

[35] So H-C, Chau K-L, Ao F-K, Mo C-H, Sham P-C. Exploring shared genetic bases and causal relationships of schizophrenia and bipolar disorder with 28 cardiovascular and metabolic traits. Psychol Med 2018, in press. 10.1017/S0033291718001812 30045777

[36] Manu P, Dima L, Shulman M, Vancampfort D, De Hert M, Correll CU. Weight gain and obesity in schizophrenia: epidemiology, pathobiology, and management. Acta Psychiatr Scand 2015; 132: 97–108. 26016380 10.1111/acps.12445

