# Supplementary material for: Polygenic risk for schizophrenia, disordered eating behaviours and body mass index in adolescents
Source: Br J Psychiatry. 2019 Jul;215(1):428–33. doi: 10.1192/bjp.2019.39 (PMC7117956; doi:10.1192/bjp.2019.39)
Supplement: Supplementary file 1 [file S0007125019000394sup001.docx]

**SUPPLEMENTAL MATERIAL**

**Supplemental Table 1: Distribution of disordered eating behaviours by gender at all three time points**

| **Time Points** | **Child’s sex** | **Disordered Eating behaviours** | | | |
| --- | --- | --- | --- | --- | --- |
|  |  | **Binge-eating**  **N^a^**  **n (%)** | **Purging**  **N^a^**  **n (%)** | **Fasting**  **N^a^**  **n (%)** | **Excessive Exercise**  **N^a^**  **n (%)** |
| **Age 14 years** | **Boys** | 11 (0.6%) | 10 (0.6%) | 40 (2.3%) | 31 (1.8%) |
|  | **Girls** | 37 (1.7%) | 52 (2.5%) | 200 (9.4%) | 49 (2.3%) |
| **Age 16 years** | **Boys** | 10 (0.7%) | 17 (1.2%) | 40 (2.9%) | 28 (2.1%) |
|  | **Girls** | 114 (5.9%) | 180 (9.4%) | 399 (20.9%) | 60 (3.1%) |
| **Age 18 years** | **Boys** | 9 (1.1%) | 14 (1.7%) | 35 (4.2%) | 14 (1.7%) |
|  | **Girls** | 88 (6.2%) | 127 (9.1%) | 226 (16.1%) | 69 (4.9%) |

**Supplemental Figure 1: Association between SCZ PRS derived across SNPs p-value thresholds and binge eating**

**Supplemental Figure 2: Association between SCZ PRS derived across SNPs p-value thresholds and BMI**
